# Mapping quality of primary care among 0.5 million people with multiple long-term conditions in England, 2015–2024: a retrospective cohort study using electronic health records

**DOI:** 10.1186/s12916-026-04964-z

**Published:** 2026-07-10

**Authors:** Qian Gao, Meryem Cicek, Benedict Hayhoe, Geva Greenfield, Michaela Otis, Gesthimani Misirli, Azeem Majeed, Paul Aylin, Alex Bottle

**Affiliations:** 1 School of Public Health, Imperial College London, London, UK; 2 Faculty of Population Health Sciences, University College London, London, UK

**Keywords:** quality of care, primary care, multiple long-term conditions, England

## Abstract

**Background:**

As the number of people with multimorbidity continues to increase, the traditional medical care model – based on single disease models - has become increasingly unsuitable to meet current challenges, leading to concerns about the quality of primary care for patients with multimorbidity. This study aimed to evaluate the quality of care for people with multimorbidity in primary care, using quality indicators identified in Electronic Health Records (EHR) data.

**Methods:**

This study used EHR from the Discover-NOW Hub for April 2015–2024, covering 95% of northwest London’s population. Through review of literature and clinical guidelines, we identified eight generic and six multimorbidity-specific quality indicators (QIs) for primary care, with consideration of the availability of indicators within EHR data. The final list was derived following discussion with people with lived experience of multimorbidity and clinicians. We used heat maps to report the QI attainment levels and conducted Ordinary Least Squares and logistic regression analyses to assess sociodemographic and health-related inequalities in relation to generic and multimorbidity-specific QI.

**Results:**

This study included 509,782 adult patients with multimorbidity. Attainment on generic QIs (range 23.5%-79.8%) was generally higher than multimorbidity-specific indicators (< 30%). QIs were generally stable over the past decade, but significantly reduced during the COVID-19 pandemic and fluctuated across individual QIs. Compared with healthier multimorbidity groups, those living with more comorbidities, polypharmacy and more severe frailty had higher levels of QI scores and had higher odds of uptake of individual QIs. Notably, we found socioeconomic inequalities, especially regarding ethnicity and neighbourhood deprivation, though this varied by QI.

**Conclusions:**

This study provides real-world evidence of the quality of primary care among multimorbidity patients. There was lower attainment on multimorbidity-specific QI than general indicators, with less affluent and some minority ethnic groups less likely to receive good care. Policymakers should focus on approaches to improve the support of primary care clinicians in the delivery of person-centred care for individuals with multimorbidity. Further research is needed to explore the underlying mechanisms for differential QI attainment and support primary care teams to improve the quality of care for people with multimorbidity.

**Supplementary Information:**

The online version contains supplementary material available at https://doi.org/10.1186/s12916-026-04964-z.

## Background

The care needs of individuals with multimorbidity, defined as those with co-existence of two or more chronic conditions [1], are often complex in terms of the management and treatment of multiple conditions together [2, 3]. The interplay of diseases may worsen patients’ existing health conditions and complicate healthcare regimens [4], requiring attention to optimise and personalise care provision in a tailored and timely manner. These patients often require input from multiple healthcare specialities and have multiple interactions with different healthcare teams. Navigating such complex care can be hard for both patients and healthcare professionals [5]. Having a greater number of comorbid conditions is associated with poorer health-related quality of life [6], and increased mortality risk [7], emphasising the need for high-quality care delivery.

Although there are various ways of characterising and measuring multimorbidity [8, 9], there is limited literature on different approaches to measuring the quality of care specific to multimorbidity. The World Health Organization (WHO) [2, 10] defines the core domains of quality of care in general, including effectiveness, safety and patient-centred care, which should be prioritised through timely, equitable, integrated and efficient care delivery. Some other domains in care coordination, shared decision-making and patient satisfaction are particularly crucial for multimorbidity care [2]. A recent scoping systematic review [11] outlined key domains for assessing the quality of care for multimorbidity: (i) *healthcare utilisation* (type, frequency, length); (ii) *the process of care* (continuity, coordination, comprehensiveness, patient-centredness, management of lifestyle factors, management of specific diseases and medicines); (iii) *patient experience and satisfaction*; and (iv) *outcomes of care* (patient-reported outcomes, health-related quality of life, adverse events).

Much of traditional medical care is built on a single-disease focused model. This means that clinical guidelines and recommendations for best medical practice have also tended to be single-disease focused [12], which become less suitable as multimorbidity, including mental-physical health multimorbidity, continues to grow in prevalence [13]. Traditional quality measures, often derived from clinical guidelines, tend also to focus on single conditions and may be inadequate for assessing care of patients with multimorbidity [14]. Furthermore, current multimorbidity quality indicator (QI) studies tend to focus more on older [15] or highly complex patients [16] and may not comprehensively capture the full diversity of multimorbidity patients (e.g. different age groups, and a wide range of health conditions in relation to multimorbidity). There is limited quantitative evidence on the level and inequality of quality of care in patients with multimorbidity. For example, previous research has relied on small samples primarily for measurement validation [17], which may not comprehensively reflect the quality of care for patients with multimorbidity. In addition, little quantitative evidence specifically targets the quality of primary care for this patient group.

To improve standards, it is fundamental to measure quality of care in tangible terms and integrate a whole systems-based approach to deal with fragmented care of multimorbidity at macro, meso and micro system levels, with a particular focus on enhancing the quality of primary care services [10]. Combining evidence-based prior indicators and context-specific additions based on input from both patients and experts has been suggested as an optimal approach for developing an applicable set of QIs [11, 15, 16, 18]. This requires integration of general recommendations into tangible metrics tailored to the context of the population and available data sources. It is key to involve assessing the utility of existing quality of care measures in routine datasets. For example, the quality outcomes framework (QOF) a national scheme that provides financial incentives to GP practices in the management of their patients, which collects routine data in primary care as indicators for quality of disease and medicine management and healthcare utilisation [19]. Although no QOF indicators existed specific to multimorbidity, the framework includes conditions that are highly prevalent in multimorbidity and their associated indicators.

To address current evidence gaps, this study aimed to evaluate the quality of care for people with multimorbidity in primary care, using quality indicators identified in Electronic Health Records (EHR) data.

## Methods

### Data design and population

The study used the deidentified dataset from the Discover-NOW Hub’s Whole Systems Integrated Care (WSIC) database for the diverse population in Northwest London. It includes pseudonymised and linked routinely collected patient data across NHS primary care, secondary care, mental health, and community services from Jan 2015 onwards [20] and covers 95% of the total northwest London population. The NOW WSIC databases received ethical approval from the UK Health Research Authority (reference 23/WM/0196).

### Procedures

This study included adults with multimorbidity, defined as two or more of an identified set of long-term conditions, registered with a GP in Northwest London, and with active GP records (defined as with GP records during the corresponding financial year) between 1st April 2015 and 30th April 2024. We prioritised UK National Institute for Health and Care Excellence (NICE) guidelines as a key grounding reference for QI [21], and we identified an initial list of long-term conditions outlined in the 2021/22 UK Quality and Outcomes Framework (QOF) [19]. Working with patient representatives and clinicians, we identified a set of long-term conditions relevant to multimorbidity in the WSIC data *(Additional File: Table **S1*). We initially identified yearly cohorts of adult patients with multimorbidity according to their number of long-term conditions by the end of each financial year (2015–2024). We then extracted the health records of each cohort for the year after they were identified as having multimorbidity to generate the final cohorts. Details of study samples are in *Additional File: Table S2*.

### Measures

#### Outcomes: quality indicators of primary care

We explored and identified evidence-based markers of quality of care for people with multimorbidity by NICE guidelines [21]. We included indicators relating to multimorbidity risk factors, complications, and definitions from NICE guidance and QOF indicators for conditions highly prevalent in multimorbidity. We focused on indicators relating to primary care processes or interventions (e.g. blood pressure measurement) rather than clinical outcomes or care coordination. We defined and reviewed the resulting indicators with public partners who live with multimorbidity and are part of our multimorbidity and mental health research team to gauge the suitability and relevance to patients and their care of proposed indicators. Eight public partners contributed across four consecutive videoconference meetings (study conception, initial search, extended search and final list of indicators). In parallel, working with our panel of researchers and clinicians (one senior consultant and one GP), we refined this list further based on what is feasible, measurable and reliably recorded in the WSIC database, through consultation with WSIC data managers (*Additional File: Table S3-4*).

This produced two final sets of indicators, (1) generic clinical indicators and (2) multimorbidity-specific indicators, for which we retrieved the Systematized Medical Nomenclature for Medicine–Clinical Terminology (SNOMED CT) clinical codes used by UK NHS Digital. The first set includes indicators related to important clinical monitoring checks likely to be relevant to all patients but of potentially greater importance in individuals with multimorbidity, including: blood pressure, serum cholesterol, blood sugar levels, smoking, body mass index (BMI), annual influenza vaccination, alcohol consumption and medication reviews. The second set included primary care checks more specific to individuals with multiple long-term conditions: polypharmacy reviews, frailty and functional assessment, reviewing the risk of hospital admission, review of care plans, mental health screening (depression/anxiety), and reviewing the risk of falls. The SNOMED CT clinical code lists for each QI were clinically reviewed to confirm accuracy and relevance. Finally, based on these SNOMED CT code lists, we generated records for our QIs in the primary care health records for the identified multimorbidity cohorts in WSIC data. Indicators were recorded annually as binary (1 = yes, 0 = no), and composite QI scores were calculated by summing up responses of each indicator on an individual level, with scores ranging from 0 to 14.

### Covariates

In coordination with the Vector Model of Patient Complexity [22], we considered patients’ demographic characteristics (age, sex [female vs. male], ethnicity [White, Asian, Black/African-Caribbean, mixed/others]) and Index of Multiple Deprivation (IMD) [23] in our analysis. IMD provides the relative socioeconomic deprivation of small geographical areas according to the patient’s residential location, and we used IMD deciles as a continuous measure in the modelling. We considered health-related factors in the last 12 months, such as the number of comorbidities, frailty and polypharmacy. Frailty was measured and documented using the validated electronic frailty index (eFI), which consists of 36 health deficits and categorises individuals as being ‘fit’, or having ‘mild’, ‘moderate’, or ‘severe frailty’. Polypharmacy is defined as the prescription of five or more prescribed medications in the past 12 months and is also a component of eFI [24].

### Statistical analysis

We measured the yearly level of attainment on each QI. We then assessed sociodemographic factors and health inequalities in overall scores of primary care QI among multimorbidity patients, as well as whether and how individual generic and multimorbidity-specific QI drive the associations. Using the latest year (2023-24), we used Ordinary Least Squares (OLS) regression for the composite QI score. In diagnostics checks for the assumption of OLS regression models, the distribution of residuals was close to homoscedastic and normal for fully adjusted models. There was a low risk of multicollinearity, with mean variance inflation factor (VIF) of at most 1.78 amongst independent variables. We then applied logistic regression models assessing whether the level of individual generic and multimorbidity-specific QI varied by patients’ demographic and health-related characteristics. In sensitivity analyses, we further added the interactions between age and the number of comorbidities to the fully adjusted models. All OLS regression and logistic regression models were replicated in the 2018–2019 pre-Covid cohort. A 2-sided p-value of less than 0.05 was considered statistically significant for all analyses as this was an exploratory rather than hypothesis-testing study. All analyses were performed using R studio and Stata 16.0.

## Results

The study cohorts ranged from 268,844 (2015-16) to 509,782 (2023-24), with nearly half of included individuals aged 60 years and above. In the latest year (2023-24), the average age was 56.8 years, 54.6% were female, and 46.2% were White adults. For health conditions, 66.7% had two comorbidities, 13.5% had four or more comorbidities, 32.6% had polypharmacy in the past 12 months, and about half had mild to severe frailty. Hypertension and diabetes were the commonest conditions. The median score of QI was 4.0 (IQR 2–6), with a median score of generic indicators at 4.0 (IQR 2–5) and multimorbidity-specific indicators at 0 (IQR 0–1) (Table 1*& Additional File: Table S5*).

**Table 1:** Patient demographic and health characteristics

Characteristics	2023-24 (*n* = 509,782)
**Registered patients**	1,982,427
**Age (Mean**,** SD)**	56.8 (17.8)
< 30 years	41,845 (8.2%)
30–60 years	231,276 (45.4%)
≥ 60 years	236,661 (46.4%)
**Gender**	
Female	278,240 (54.6%)
Male	231,516 (45.4%)
Missing	26 (0.01%)
**Ethnicity**	
White	235,267 (46.2%)
Asian	147,883 (29.0%)
Black/African-Caribbean	57,720 (11.3%)
Others (inc. mixed ethnicity)	66,682 (13.1%)
Missing (inc. not known/stated)	2,230 (0.4%)
**Index of multiple Deprivation** (Deciles)	
Most deprived decile	14,410 (2.8%)
2nd	12,204 (2.4%)
3rd	61,820 (12.2%)
4th	80,472 (15.8%)
5th	86,908 (17.1%)
6th	76,595 (15.0%)
7th	64,370 (12.6%)
8th	44,008 (8.6%)
9th	40,252 (7.9%)
Least deprived decile	27,560 (5.4%)
Missing	1,183 (0.2%)
**Multimorbidity** (median, IQR)	2 (2–3)
**Number of comorbidities** (range)	(2–14)
Total with 2 morbidities	339,811 (66.7%)
Total with 3 morbidities	101,246 (19.8%)
Total with 4 or more morbidities	68,725 (13.5%)
**Hypertension**	233,359 (45.8%)
**Diabetes**	136,198 (26.7%)
**Polypharmacy (past 12 months)**	
yes	166,278 (32.6%)
no	325,164 (63.8%)
missing	18,340 (3.6%)
**Frailty index categories**	
Fit	225,240 (44.2%)
Mild	124,665 (24.5%)
Moderate	83,193 (16.3%)
Severe	58,344 (11.4%)
Missing	18,340 (3.6%)
**QI scores (Median**,** IQR)**	
Total QI scores (ranged 0–14)	4 (2–6)
Generic indicators scores (ranged 0–8)	4 (2–5)
Multimorbidity-specific indicators scores (ranged 0–6)	0 (0–1)

**Fig. 1:**
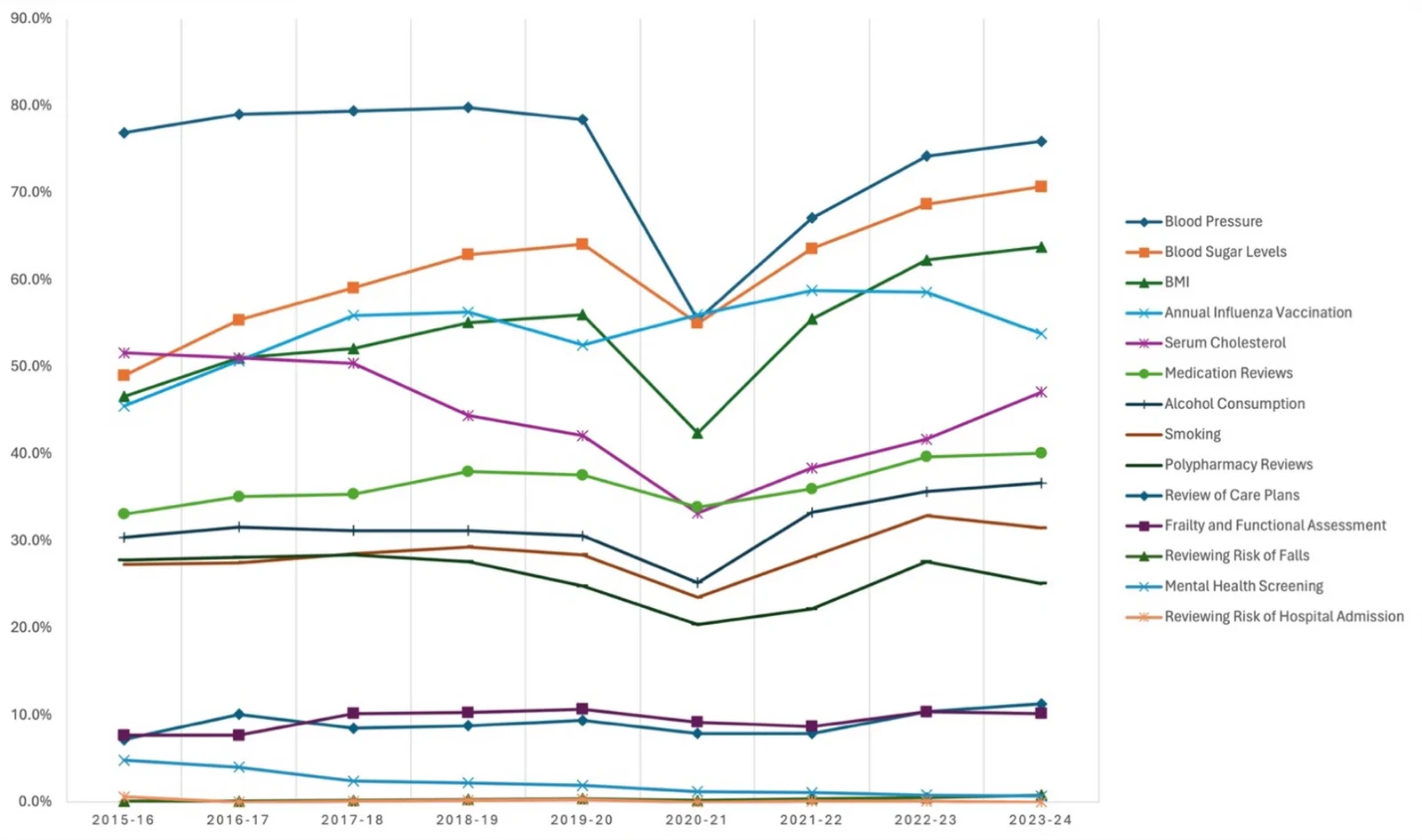
The changes in Quality Indicator attainment among multimorbidity patients in Northwest London over 2015-24

Over 2015-24, the yearly level of attainment of QI was generally stable, except for significant reductions for all QI during the COVID-19 pandemic period (Fig. 1*& Additional File: Table S6*). Table 2 shows the attainment over 2015-24. For generic indicators, the highest engagement was blood pressure checks (55.4%-79.8%), followed by health checks for blood glucose levels (49.0%-70.7%), BMI (42.4%-63.8%), annual influenza vaccination (45.5%-58.8%), and serum cholesterol (33.2%-51.6%). The level of attainment on medication reviews (33.1%-40.1%), health checks for alcohol consumption (25.2%-36.7%) and smoking status (23.5%-32.9%) were lower. Attainment on multimorbidity-specific indicators was generally lower than generic indicators, with polypharmacy reviews at the top (20.4%-28.4%), followed by review of care plans (7.2%-11.3%), and frailty and functional assessment (7.7%-10.7%). There was lower than 5% attainment on the remaining indicators (mental health screening, reviews of hospital admission and risk of falls).

**Table 2:** The attainment level of quality Indicators among multimorbidity patients in Northwest London (2015-24)

### Quality indicator scores

Multivariable OLS regression showed strong associations between sociodemographic and health factors with QI scores: older age, males, and those living with greater number of comorbidities, polypharmacy and higher frailty levels were associated with increased levels of overall and generic QI scores. Compared with White adults, those of Asian, Black/African-Caribbean and mixed/others ethnicity had increased levels of overall and generic QI scores, but Asian and mixed/other ethnicity had slightly decreased levels of multimorbidity-specific QI scores. While less-deprived deciles were associated with lower overall levels of QI scores and generic QI scores, there was no statistical evidence of association between deprivation and multimorbidity-specific QI scores (Table 3).

**Table 3:** Associations between sociodemographic and health characteristics and scores of quality indicators (2023-24)

Characteristics	Overall QI scores (Coef., 95%CI)	*P* value	Generic QI Score (Coef., 95%C)	*P* value	Multimorbidity specific QI Score (Coef., 95%C)	*P* value
**Age** (ref. < 30 years)						
30–60 years	1.15 (1.12–1.17)	< 0.001	1.12 (1.10–1.14)	< 0.001	0.03 (0.02–0.04)	< 0.001
≥ 60 years	1.75 (1.72–1.78)	< 0.001	1.62 (1.60–1.65)	< 0.001	0.13 (0.12–0.13)	< 0.001
**Gender** (ref. female)						
Male	0.40 (0.39–0.41)	< 0.001	0.37 (0.36–0.38)	< 0.001	0.03 (0.02–0.03)	< 0.001
**Ethnicity** (ref. White)						
Asian	0.30 (0.29–0.32)	< 0.001	0.33 (0.32–0.34)	< 0.001	-0.03 (-0.03 - -0.02)	< 0.001
Black/African-Caribbean	0.16 (0.14–0.18)	< 0.001	0.16 (0.14–0.18)	< 0.001	0.002 (-0.004–0.01)	0.56
Others	0.10 (0.08–0.12)	< 0.001	0.12 (0.11–0.14)	< 0.001	-0.02 (-0.03 - -0.02)	< 0.001
**IMD**	-0.04 (-0.04 - -0.03)	< 0.001	-0.04 (-0.04 - -0.03)	< 0.001	>-0.001 (-0.001- 0.001)	0.36
**Number of comorbidities** (ref. 2 morbidities)						
3 morbidities	0.69 (0.68–0.71)	< 0.001	0.62 (0.60–0.63)	< 0.001	0.07 (0.07–0.08)	< 0.001
4 or more morbidities	1.03 (1.01–1.05)	< 0.001	0.88 (0.86–0.89)	< 0.001	0.15 (0.14–0.16)	< 0.001
**Polypharmacy** (ref. no)	0.59 (0.58–0.61)	< 0.001	0.50 (0.49–0.52)	< 0.001	0.09 (0.08–0.09)	< 0.001
**Frailty** (ref. fit)						
Mild	0.80 (0.78–0.82)	< 0.001	0.66 (0.65–0.68)	< 0.001	0.14 (0.13–0.14)	< 0.001
Moderate	1.18 (1.16–1.20)	< 0.001	0.78 (0.76–0.80)	< 0.001	0.39 (0.39–0.40)	< 0.001
Severe	1.66 (1.63–1.69)	< 0.001	0.76 (0.74–0.79)	< 0.001	0.90 (0.89–0.90)	< 0.001

Note. Models adjusted for age, gender, ethnicity, IMD, number of comorbidities, polypharmacy (past 12 months), and frailty index

### Generic indicators

**Fig. 2:**
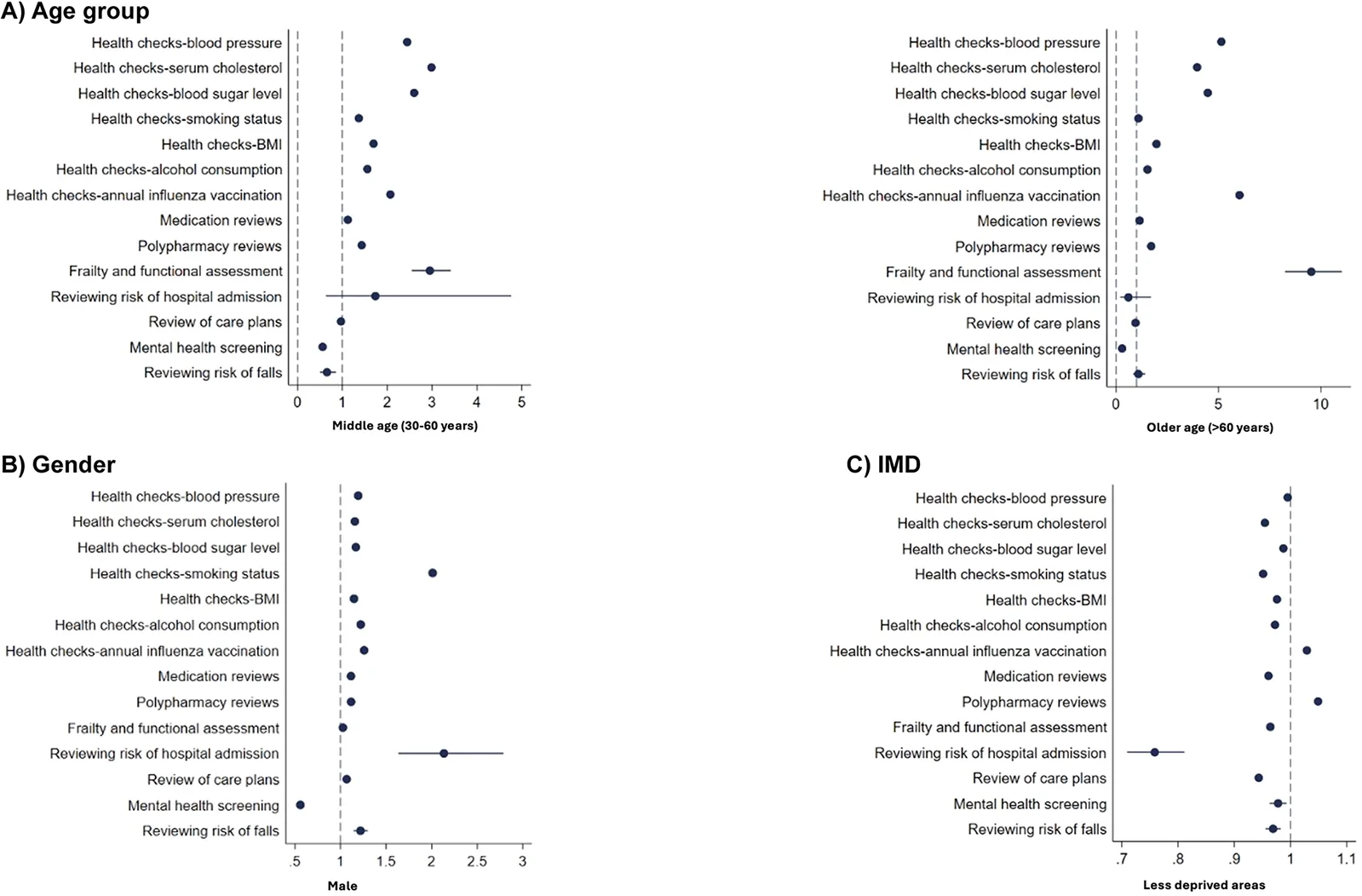

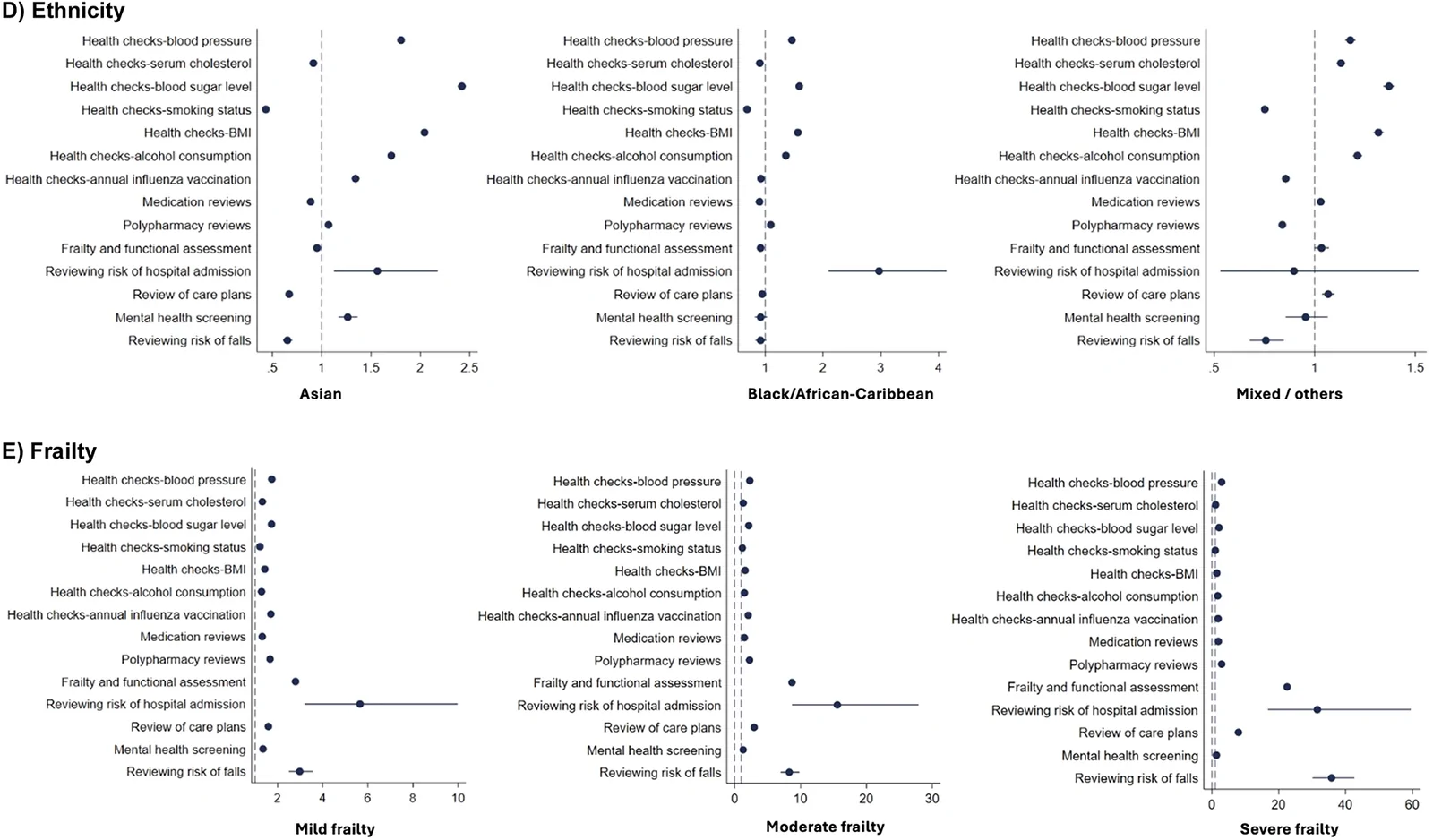

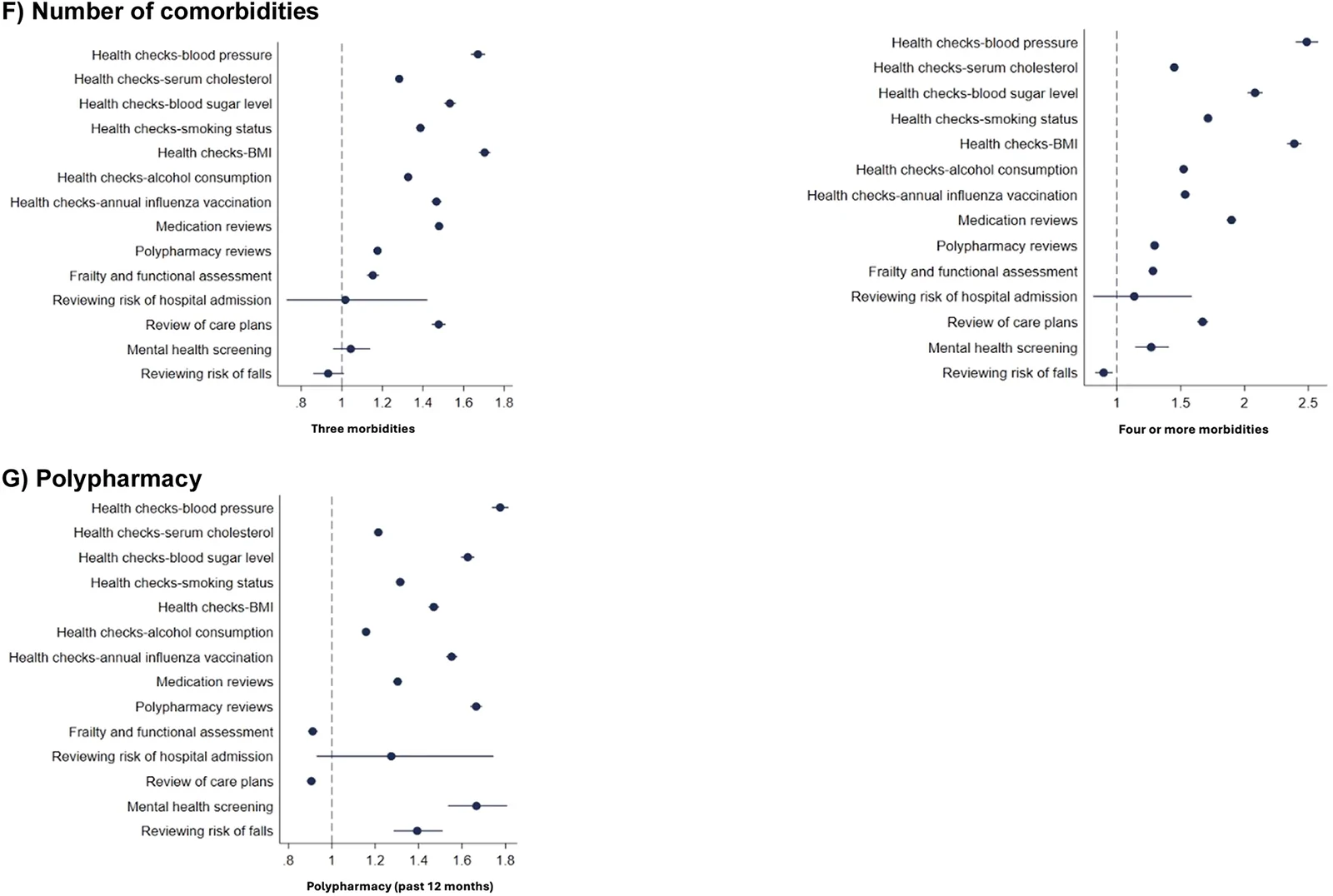
Adjusted odds ratios from regression models testing sociodemographic and health inequalities in individual generic and multimorbidity-specific quality indicators (2023-24). Note: (**A**) Age group: reference category < 30 years; (**B**) Gender: reference category female; (**C**) IMD: reference category more deprived; (**D**) Ethnicity: reference category White; (**E**) Frailty: reference category fit; (**F**) Number of comorbidities: reference category two morbidities; (**G**) Polypharmacy: reference category no

Multivariable regression models showed that, compared with younger adults (< 30 years), middle-aged and older adults had higher odds of all generic indicators (Fig. 2*& Additional File: Table S7*). The odds of generic QIs were higher for males than females. There were mixed findings for ethnicity. Compared with White adults, those of Asian, Black, mixed and other ethnicities had higher odds of recorded checks for blood pressure, BMI, blood sugar levels and alcohol consumption, but generally had lower odds of serum cholesterol, smoking, annual influenza vaccination and medication reviews. Compared with more deprived IMD deciles, less-deprived groups were associated with lower odds of generic QI except for annual influenza vaccination (OR = 1.03, 95%CI 1.02–1.03). Increased number of comorbidities, polypharmacy and more severe frailty were consistently associated with increased odds of all generic QIs than those healthier groups with no polypharmacy or no frailty.

### Multimorbidity-specific indicators

Findings for multimorbidity-specific indicators were mixed across indicators (Fig. 2*& Additional File: Table S8*). Middle-aged and older adults were more likely to receive polypharmacy reviews and functional assessment but had lower odds of mental health screening than younger people. Odds were higher for men than women across all multimorbidity-specific indicators except for mental health screening. Compared with those of white ethnicity, Asian and Black ethnicity had higher odds of polypharmacy reviews, reviewing risk of hospital admission, but lower odds of functional assessment and reviewing care plans. Asian adults also had higher odds of mental health screening and lower odds of reviewing risks of falls. Those of mixed/other ethnicity had decreased odds of polypharmacy reviews and reviewing risk of falls, and increased odds of functional assessment and reviews of care plans compared with White adults, although the difference was small. Less deprivation was associated with lower odds of all multimorbidity-specific indicators except for polypharmacy reviews than more deprived groups. Compared with those with only two long-term conditions, the odds of multimorbidity-specific indicators were generally higher for those with more than two comorbid conditions, although the odds of reviewing risk of falls were lower for those with four or more morbidities. Compared with fit individuals, more severe frailty was associated with increased odds of all multimorbidity-specific indicators. Individuals with polypharmacy had increased odds of polypharmacy reviews, mental health screening and reviewing risk of falls, with lower odds of functional assessment and care plans reviews than non-polypharmacy groups.

### Sensitivity analyses

In sensitivity analyses, we added the interactions between age and number of comorbidities in the above models. Results did not differ from the original models, with a slight increase in odds ratios for age, polypharmacy, and frailty. Using the pre-COVID 2018-19 cohort, we additionally conducted sensitivity analyses for the above logistic regression models assessing associations between sociodemographic and health characteristics and QI (attainment on individual indicators). The results of sensitivity analyses were broadly consistent with the findings in man analyses of the post-COVID 2023-34 cohort (*Additional File: Table S9-10*).

## Discussion

This study provides real-world evidence on the quality of primary care for multimorbidity patients using EHRs. Attainment on generic QI (up to 80%) was higher than multimorbidity-specific indicators (< 30%). Quality was generally stable over the past decade, but with significant reductions for all QIs during the COVID-19 pandemic. In general, those with greater health needs had higher levels of the composite QI score and were more likely to have individual QIs met. There was evidence of some socioeconomic inequalities, although findings were mixed.

Attainment on generic QIs was nearly double that of multimorbidity-specific QIs, although there were variations across indicators. Notably, attainment on some multimorbidity-specific indicators (mental health screening, reviews of hospital admission and risk of falls) was relatively low. Suboptimal performance in these key areas may pose risks to patient safety and suggest potential gaps in care quality, highlighting the need further to optimise essential care processes and ensure appropriate clinical review for patients with complex health needs. Moreover, these low attainment multimorbidity-specific QIs are often more time-consuming to implement compared with generic indicators. Strengthening these QIs through digital technologies is essential to advancing future care programs. Quality of care for multimorbidity is influenced by comorbidities and health-seeking behaviours and the infrastructure surrounding integrated patient care for people with multimorbidity [4]. In general, we found that quality was stable over the past decade but with a dip in all indicators associated with the COVID-19 pandemic period, as has been found for many single conditions [25]. This could be largely due to the reduction in face-to-face consultations in general practice during the pandemic, as well as the temporary removal or reduction of financial incentives for many of our indicators in QOF implementation [25]. This highlights the crucial role of macro-level health policy in affecting the quality of care [10].

Theoretically, improving the quality of care can help address social determinants of health [26]. However, we found that after accounting for health factors, there were ethnicity-related inequalities in quality of care, although findings were mixed across individual QIs. Specifically, we found that compared with White patients, those of Asian, Black/African-Caribbean, and mixed/other ethnicity had higher overall and generic QI scores, but Asian and mixed/other ethnicity generally had lower multimorbidity-specific QI scores. This aligns with previous evidence that the delivery of potentially beneficial clinical procedures is likely to vary across socioeconomic and ethnicity [27].

Although previous research (using 2008-18 Hospital Episode Statistics data) reported that people living in more affluent areas of England receive higher quality of care [28], our findings suggest that those living in less-deprived areas had lower odds of uptake of clinical checks for the majority of generic indicators except for annual influenza vaccination and polypharmacy reviews. These findings were generally in line with previous evidence of the narrowing inequalities in healthcare quality (especially for patient experience and access to healthcare) in England over the past decade [29]. The socioeconomic inequalities in QI could potentially be driven by health inequalities by deprivation. The estimated number of adults with major illnesses in the most-deprived areas was over double that of least-deprived areas in England [30]. Thus, the higher QI in less-deprived groups may be partially attributable to the profiles and severity of multimorbidity across people living in different deprivation areas, although we adjusted for the number of conditions and frailty [11]. On the supply side, deprivation inequalities in quality of care may be shaped by practice-level indicators across area-level deprivation, which warrants further investigation.

Best practice for multimorbidity management in primary care is not one-size-fits-all, and the complexity of many patients’ multimorbidity is likely to be associated with more complex care needs, as well as greater challenges in terms of healthcare navigation and engagement. The complex and heterogeneous health needs associated with multimorbidity may lead to a mismatch in healthcare provision, resulting in under-served patient groups and reduced quality of care [31]. When making a treatment plan for patients with complex health needs, clinicians may make trade-offs with care processes, potentially leading to the overuse or underuse of certain interventions, especially in the absence of well-suited clinical guidelines [22].

Notably, poorer health states—such as having more comorbidities, polypharmacy, and higher degrees of frailty—were consistently associated with higher achievement of quality indicators. However, achievement of generic quality indicators was generally much higher than multimorbidity-specific indicators. This may partially be due to the nature of the indicators that we identified [11]. Generic indicators mostly involve quite straightforward processes or interventions (e.g. blood pressure checks, blood tests for blood sugar and cholesterol), which may be conducted routinely or opportunistically as part of a wide range of healthcare interactions. Sicker individuals with multimorbidity will attend GP appointments more frequently, with consequently more opportunity for ‘ticking off’ these checks. Multimorbidity-specific indicators we identified were generally more complex (e.g. frailty assessment, care planning) and consequently likely to require more time and specific focus of healthcare professionals; this may be reflected in the dip in achievement of the more straightforward, practical, generic indicators during the pandemic period not being seen in the multimorbidity-specific indicators. This argues for more specific focus on individuals with multimorbidity and their care, with time allocated for these more complex processes.

The findings highlight the necessity of improving quality of primary care for supporting multimorbidity management in the community as well as reducing socioeconomic inequalities in quality of care [31]. The sociodemographic inequality in quality raises the potential risks of affecting equality in long-term health outcomes. Future studies could explore the underlying reasons and mechanisms for the low rate of these multimorbidity-specific indicators and variations in them by patient factors and wider determinants. Moreover, further research needs to investigate effective QIs for supporting person-centred multimorbidity management in primary care, for example, providing insights into the relation between QIs and health demands and health outcomes over time. This will have practical applications for local policymakers to identify priority areas to optimise health services and improve quality of care for these patients with greater health needs.

This study provides the first estimates of the yearly level of attainment on QI for patients with multimorbidity in UK’s primary care over the past decade. Our QIs are evidence-based and have been agreed using inputs from key stakeholders, with a view to finding ways in which care for people with multimorbidity might be improved. Another key strength is the benefits of large EHRs that provide rich information on attainment of QI in primary care. The comprehensive list of SNOMED CT clinical codes enables us to conceptualise and measure QI in primary care and explore sociodemographic and health inequalities in quality of care. Analysis of EHR data of course cannot include what has not been recorded. Further, we cannot rule out the possibility of the low attainment rate partially impacted by the SNOMED CT clinical codes adopted in the study, and quantitative investigations could further validate our findings. We measured overall, generic and multimorbidity-specific scores and captured the individual items of QI to limit the potential bias introduced using overall scores. Our study considered the number of comorbidities but not the complex patterns of multimorbidity, so further study is needed to explore the performance across different multimorbidity groups, especially for those discordant clusters. In confounder selection, we considered the Vector Model of Patient Complexity [22] and there is the possibility of residual confounding due to the availability of variables in EHRs. Further, variations in the associations across individual indicators may be affected by statistical power (low attainment on some multimorbidity-specific indicators).

## Conclusions

We developed and measured evidence-based quality of care for multimorbidity patients in NW London using a set of process indicators in large linked EHR data. The level of quality of primary care for multimorbidity patients was stable over the past decade, bar a dip during the COVID-19 pandemic’s peak. Attainment on generic quality indicators was higher than multimorbidity-specific indicators but varied across indicators. Policy makers should consider increased resource for primary care to support the provision of care specifically focused on the more complex, time-consuming interventions that indicate quality of care for individuals with multimorbidity. There were some socioeconomic inequalities, which, while mixed, may suggest scope for primary care clinicians to improve the quality of care of individuals with multimorbidity, and of those from less affluent and minority ethnic backgrounds specifically. Further research is needed to explore the underlying mechanisms for discrepancies in QI attainment and better support clinicians managing this patient group.

## Supplementary Information

Below is the link to the electronic supplementary material.

Supplementary Material 1: Table S1–Table S10

## Data Availability

The NOW Whole System Integrated Care dataset (WSIC) was available through monitored request and approved by the Northwest London Data Access Committee (DAC) (https://discover-now.co.uk/).

## Abbreviations

QIs: Quality indicators
WHO: World Health Organization
WSIC: Whole Systems Integrated Care
QOF: Quality and Outcomes Framework
SNOMED CT: Systematized Medical Nomenclature for Medicine–Clinical Terminology
BMI: Body mass index
IMD: Index of Multiple Deprivation
eFI: Electronic frailty index
OLS: Ordinary Least Squares
VIF: Variance inflation factor
