# Supplementary material for: Mapping quality of primary care among 0.5 million people with multiple long-term conditions in England, 2015–2024: a retrospective cohort study using electronic health records
Source: BMC Med. 2026 Jul 10;24:551. doi: 10.1186/s12916-026-04964-z (PMC13644081; doi:10.1186/s12916-026-04964-z)
Supplement: Supplementary file 1 [file 12916_2026_4964_MOESM1_ESM.docx]

**Additional File 1**

**Table S1.** Lists of long-term conditions

**Table S2.** Selection of study samples

**Table S3.** Initial list of quality of care indicators in primary care for patients with multimorbidity

**Table S4.** Final list of quality of care indicators in primary care for patients with multimorbidity

**Table S5.** Patient demographic and health characteristics

**Table S6.** The levels of quality Indicators among multimorbidity patients in Northwest London (2015-24)

**Table S7.** Sociodemographic and health inequalities in individual generic quality indicators (2023-24)

**Table S8.** Sociodemographic and health inequalities in individual multimorbidity specific quality indicators (2023-24)

**Sensitivity analysis**

**Table S9.** Sociodemographic and health inequalities in individual generic quality indicators (2018-19)

**Table S10.** Sociodemographic and health inequalities in individual multimorbidity specific quality indicators and overall scores of quality indicators (2018-19)

**Table S1. Lists of long-term conditions**

|  | **Long-term conditions** |
| --- | --- |
| 1 | asthma |
| 2 | anxiety |
| 3 | atrial fibrillation |
| 4 | cancer |
| 5 | coronary heart disease [CHD] / ischaemic heart disease |
| 6 | chronic kidney disease [CKD] |
| 7 | chronic obstructive pulmonary disease [COPD] |
| 8 | dementia |
| 9 | depression |
| 10 | diabetes |
| 11 | epilepsy |
| 12 | heart failure |
| 13 | hypertension |
| 14 | mental health [i.e. severe mental illness such as schizophrenia, bipolar affective disorder and other psychoses] |
| 15 | obesity |
| 16 | osteoporosis |
| 17 | peripheral arterial disease [PAD] |
| 18 | rheumatoid arthritis |
| 19 | stroke/ transient ischaemic attack [TIA] |

**Table S2. Selection of study samples**

| **Years** | **2015-16** | **2016-17** | **2017-18** | **2018-19** | **2019-20** | **2020-21** | **2021-22** | **2022-23** | **2023-24** |
| --- | --- | --- | --- | --- | --- | --- | --- | --- | --- |
| GP Registered patients | 1,563,027 | 1,409,247 | 1,423,919 | 1,479,277 | 1,537,030 | 1,832,409 | 1,940,563 | 1,944,423 | 1,982,427 |
| Multimorbidity patients | 273,557 | 293,473 | 321,176 | 346,567 | 373,725 | 396,630 | 356,635 | 475,477 | 517,636 |
| Multimorbidity patients with GP records | 268,844 | 285,136 | 311,666 | 336,693 | 363,197 | 390,307 | 352,779 | 468,172 | 509,782 |

** age >18 years ** Registered patients in NW primary practices*

**Table S3. Initial list of quality of care indicators in primary care for patients with multimorbidity**

| **Indicator Category** | **Sub-category** | **Indicator** | **SNOMED Clinical Term** |
| --- | --- | --- | --- |
| Baseline clinical checks | Blood pressure | Has the patient's blood pressure been checked in the past 12 months | Blood pressure taking assessment (procedure) |
|  |  |  | Measurement of blood pressure in left arm (procedure) |
|  |  |  | Measurement of blood pressure in right arm (procedure) |
|  | Serum cholesterol | Has the patient's serum cholesterol been checked in the past 12 months | Cholesterol measurement (procedure) |
|  |  |  | Cholesterol content measurement (procedure) |
|  |  |  | Total cholesterol measurement (procedure) |
|  |  |  | High density lipoprotein cholesterol measurement (procedure) |
|  |  |  | Low density lipoprotein cholesterol measurement (procedure) |
|  | Blood sugar levels | Has the patient's blood sugar levels been checked in the past 12 months | Glucose measurement, blood (procedure) |
|  | Smoking | Has smoking assessment been conducted in the past 12 months | Smoking assessment (procedure) |
|  |  | Has the patient been offered smoking cessation in the past 12 months | Smoking cessation education (procedure) |
|  | BMI | Has the patient's Body Mass index been assessed in the past 12 months | Weight and body mass assessment procedure (procedure) |
|  |  |  | Measurement of body mass index (procedure) |
|  | Annual influenza vaccination | Has the patient been offered the influenza immunisation in the past 12 months | Administration of vaccine product containing only Influenza virus antigen (procedure) |
|  |  | Has the patient been offered the influenza immunisation in the past 12 months | Influenza immunisation for those in the 65 years and over and other at risk groups enhanced services administration (procedure) |
|  | Alcohol consumption | Has the patient been screened for alcohol consumption in the past 12 months | Alcohol consumption screening (procedure) |
|  |  | Has the patient been offered advice on alcohol consumption in the past 12 months | Education about alcohol consumption (procedure) |
| Reviewing **multimorbidity** severity and related risk | Polypharmacy reviews | Has the patients medications been reviewed in the past 12 months | Polypharmacy medication review (procedure) |
|  |  |  | Review of medication (procedure) |
|  |  |  | Medication review with patient (procedure) |
|  |  |  | Structured medication review (procedure) |
|  | Assessing frailty and functional assessment | Has the patient been assessed for frailty in the past 12 months | Frailty assessment (procedure) |
|  |  |  | Assessment using frailty assessment tool (procedure) |
|  |  |  | Frailty needs assessment (procedure) |
|  |  |  | Comprehensive frailty needs assessment (procedure) |
|  |  | Has the Electronic Frailty Index (eFI) been used to assess frailty in the patient in the past 12 months | Assessment using Electronic Frailty Index (procedure) |
|  |  | Has a gait speed test been performed to assess functional assessment in the patient in the past 12 months | Gait speed test (procedure) |
|  |  | Has PRISMA-7 Questionnaire been conducted to assess frailty in the patient in the past 12 months (PRISMA-7 identifies disability, score of > 3 is considered to identify frailty) | Assessment using Prisma 7 Questionnaire (procedure) |
|  | Reviewing risk of hospital admission | Has the patient been assessed for risk of hospital admission in the past 12 months | Assessment using QAdmissions emergency admission risk calculator (procedure) |
|  | Review of care plans | Has the patient's care plans been reviewed in the past 12 months | Review of care plan (procedure) |
|  |  |  | Review of personal care plan (procedure) |
|  |  |  | Review of integrated care plan (procedure) |
|  |  |  | Review of supportive care plan (procedure) |
|  | Mental health screening (Depression/anxiety) | Has the patient been screened or assessed for mental health conditions in the past 12 months | Mental health screening (procedure) |
|  |  |  | Mental health screening assessment (procedure) |
|  |  | Has the patient been screened for depression or depressive symptoms in the past 12 months | Depression screening using Patient Health Questionnaire Nine Item score (procedure) |
|  |  |  | Depression screening using questions (procedure) |
|  |  | Has the patient been screened for anxiety in the past 12 months | Anxiety screening (procedure) |
|  |  |  | Anxiety screening using questions (procedure) |
|  | Reviewing risk of falls | Has the patient been assessed for risk of falls in the past 12 months | Screening for risk of falls (procedure) |
|  |  |  | Fall risk assessment (procedure) |
|  | Non-pharmacological treatments such as diets, exercise programmes and psychological treatments | Has the patient been offered/referred a diet programme in the past 12 months | *Multiple types of diet/exercise/psychological treatments and programmes; also the difference between types of referrals to these programmes too |
|  |  | Has the patient been offered/referred an exercise programme in the past 12 months |  |
|  |  | Has the patient been referred for psychological treatment in the past 12 months |  |
|  | Healthcare utilisation | Number and type of primary care consultations patient utilises in the past 12 months | n/a (frequency) |
|  |  | Number and type of medications patient takes in the past 12 months | n/a (frequency) |
| **Indicator Category** | **Sub-category** | **Indicator** | **NICE id code (if available)** |
| **Morbidity-specific** checks (for QOF-based conditions in WSIC LTC list) | **Atrial fibrillation** | In those patients with atrial fibrillation with a record of a CHA2DS2-VASc score of 2 or more, the percentage of patients who are currently treated with anticoagulation drug therapy | NM82 |
|  |  | The percentage of patients with atrial fibrillation in whom stroke risk has been assessed using the CHA2DS2-VASc score risk stratification scoring system in the preceding 12 months (excluding those patients with a previous CHADS2 or CHA2DS2-VASc score of 2 or more) | NM81 |
|  | **Cancer** | The percentage of patients with cancer diagnosed within the preceding 15 months who have a review recorded as occurring within 3 months of the practice receiving confirmation of the diagnosis | NM62 |
|  | **Cardiovascular disease** | In those patients with a new diagnosis of hypertension or type 2 diabetes aged 25-84 years, recorded between the preceding 1 April to 31 March (excluding those with pre-existing CHD, stroke and/or TIA), who have a recorded CVD risk assessment score (using the QRISK2 assessment tool) of more than 20% in the preceding 12 months: the percentage who are currently treated with statins (unless there is a contraindication) | NM133 |
|  |  | The percentage of patients aged 25–84, years with a new diagnosis of hypertension or type 2 diabetes, recorded between the preceding 1 April to 31 March (excluding those with pre-existing CHD, stroke and/or TIA) who have had a consultation for cardiovascular risk assessment using the QRISK2 risk assessment tool between 3 months before or 3 months after date of diagnosis | NM132 |
|  | **Chronic kidney disease** | The contractor establishes and maintains a register of patients aged 18 years or over with CKD with classification of categories G3a to G5 (previously stage 3 to 5) | NM83 |
|  |  | The percentage of patients on the CKD register in whom the last blood pressure reading (measured in the preceding 12 months) is 140/90 mmHg or less | NM117 |
|  |  | The percentage of patients on the CKD register who have hypertension and proteinuria and who are currently being treated with an angiotensin-receptor blocker or an angiotensin-converting enzyme inhibitor | NM84 |
|  |  | The percentage of patients on the CKD register whose notes have a record of a urine albumin: creatinine ratio (or protein: creatinine ratio) test in the preceding 12 months | NM109 |
|  | **Chronic obstructive pulmonary disease** | The percentage of patients with COPD and Medical Research Council (MRC) Dyspnoea Scale of 3 or more at any time in the preceding 15 months, with a subsequent record of an offer of referral to a pulmonary rehabilitation programme | NM47 |
|  |  | The percentage of patients with COPD who have had influenza immunisation in the preceding 1 August to 31 March | NM106 |
|  |  | The percentage of patients with COPD with a record of FEV1 in the preceding 12 months | NM105 |
|  | **Coronary heart disease** | The percentage of patients aged 79 years or under with coronary heart disease in whom the last blood pressure reading (measured in the preceding 12 months) is 140/90 mmHg or less | NM68 |
|  |  | The percentage of patients with coronary heart disease who have had influenza immunisation in the preceding 1 August to 31 March | NM87 |
|  |  | The percentage of patients with coronary heart disease with a record in the preceding 12 months that aspirin, an alternative anti-platelet therapy, or an anti-coagulant is being taken | NM88 |
|  |  | The percentage of patients with a history of myocardial infarction (more than 12 months ago) who are currently being treated with an ACE-I (or ARB if ACE-I intolerant), aspirin (or clopidogrel) (or anticoagulant drug therapy) and a statin and a beta-blocker for those patients with left ventricular systolic dysfunction | NM80 |
|  | **Dementia (Parkinson's Disease in WSIC)** | The percentage of patients diagnosed with dementia whose care plan has been reviewed in a face-to-face review in the preceding 12 months | NM107 |
|  |  | The percentage of patients with dementia with the contact details of a named carer on their record | NM64 |
|  | **Depression** | The percentage of patients with a new diagnosis of depression in the preceding 1 April to 31 March who have been reviewed within 10-35 days of the date of diagnosis | NM50 |
|  |  | The percentage of patients with a new diagnosis of depression in the preceding 1 April to 31 March who have had a bio-psychosocial assessment by the point of diagnosis | NM49 |
|  | **Diabetes mellitus** | The contractor establishes and maintains a register of all patients aged 17 or over with diabetes mellitus, which specifies the type of diabetes where a diagnosis has been confirmed | NM41 |
|  |  | The percentage of male patients with diabetes who have a record of erectile dysfunction with a record of advice and assessment of contributory factors and treatment options in the preceding 15 months | NM52 |
|  |  | The percentage of male patients with diabetes with a record of being asked about erectile dysfunction in the preceding 15 months | NM51 |
|  |  | The percentage of patients newly diagnosed with diabetes, on the register, in the preceding 1 April to 31 March who have a record of being referred to a structured education programme within 9 months after entry on to the diabetes register | NM27 |
|  |  | The percentage of patients with diabetes who have a record of a dietary review by a suitably competent professional in the preceding 15 months | NM28 |
|  |  | The percentage of patients with diabetes who have a record of an albumin: creatinine ratio (ACR) test in the preceding 15 months | NM59 |
|  |  | The percentage of patients with diabetes who have had the following care processes performed in the preceding 12 months: BMI measurement; BP measurement; HbA1c measurement; Cholesterol measurement; Record of smoking status; Foot examination; Albumin: creatinine ratio; Serum creatinine measurement | NM74 |
|  |  | The percentage of patients with diabetes with a record of a foot examination and risk classification: 1) low risk (normal sensation, palpable pulses), 2) increased risk (neuropathy or absent pulses), 3) high risk (neuropathy or absent pulses plus deformity or skin changes or previous ulcer) or 4) ulcerated foot within the preceding 15 months | NM13 |
|  |  | The percentage of patients with diabetes with a record of testing of foot sensation using a 10g monofilament within the preceding 12 months | NM131 |
|  |  | The percentage of patients with diabetes, on the register, in whom the last blood pressure reading (measured in the preceding 12 months) is 140/80 mmHg or less | NM02 |
|  |  | The percentage of patients with diabetes, on the register, in whom the last blood pressure reading (measured in the preceding 12 months) is 150/90 mmHg or less | NM01 |
|  |  | The percentage of patients with diabetes, on the register, in whom the last IFCC-HbA1c is 64 mmol/mol or less in the preceding 12 months | NM96 |
|  |  | The percentage of patients with diabetes, on the register, in whom the last IFCC-HbA1c is 75 mmol/mol or less in the preceding 12 months | NM97 |
|  |  | The percentage of patients with diabetes, on the register, who have a record of retinal screening in the preceding 12 months | NM98 |
|  |  | The percentage of patients with diabetes, on the register, who have had influenza immunisation in the preceding 1 August to 31 March | NM139 |
|  |  | The percentage of patients with diabetes, on the register, with a diagnosis of nephropathy (clinical proteinuria) or micro-albuminuria who are currently treated with an ACE-I (or ARBs) | NM95 |
|  |  | The percentage of women with diabetes aged 17 or over and who have not attained the age of 45 who have a record of being given information and advice about pregnancy or conception or contraception tailored to their pregnancy and contraceptive intentions recorded in the preceding 12 months | NM70 |
|  | **Epilepsy** | The percentage of patients aged 18 or over on drug treatment for epilepsy who have been seizure free for the last 12 months recorded in the preceding 12 months | NM110 |
|  | **Heart failure** | The percentage of patients with heart failure diagnosed within the preceding 15 months with a record of an offer of referral for an exercise-based rehabilitation programme | NM48 |
|  | **Hypertension** | The percentage of patients aged 80 years and over with hypertension in whom the last recorded blood pressure (measured in the preceding 9 months) is 150/90 or less | NM54 |
|  |  | The percentage of patients under 80 years old with hypertension in whom the last recorded blood pressure (measured in the preceding 9 months) is 140/90 or less | NM53 |
|  |  | The percentage of patients with a new diagnosis of hypertension in the preceding 1 April to 31 March who have a record of a 12 lead ECG performed in the three months before or after the date of entry to the hypertension register | NM77 |
|  |  | The percentage of patients with a new diagnosis of hypertension in the preceding 1 April to 31 March who have a record of a test for haematuria in the three months before or after the date of entry to the hypertension register | NM76 |
|  |  | The percentage of patients with a new diagnosis of hypertension in the preceding 1 April to 31 March who have a record of urinary albumin: creatinine ratio test in the three months before or after the date of entry to the hypertension register | NM75 |
|  |  | The percentage of patients with hypertension aged 16 to 74 years in whom there is an annual assessment of physical activity, using GPPAQ, in the preceding 15 months | NM36 |
|  |  | The percentage of patients with hypertension aged 16 to 74 years who score 'less than active' on GPPAQ in the preceding 15 months, who also have a record of a brief intervention in the preceding 15 months | NM37 |
|  |  | The percentage of people with a new diagnosis of hypertension (diagnosed on or after 1 April 2014) that has been confirmed by ambulatory blood pressure monitoring (ABPM) or home blood pressure monitoring (HBPM) in the 3 months before being entered on the register | NM66 |
|  | **Hypothyroidism** | The contractor establishes and maintains a register of patients with hypothyroidism who are currently treated with levothyroxine | NM99 |
|  |  | The percentage of patients with hypothyroidism, on the register, with thyroid function tests recorded in the preceding 12 months | NM100 |
|  | **Learning disabilities** | The contractor establishes and maintains a register of patients with learning disabilities | NM73 |
|  | **Mental illness (i.e., severe mental illness)** | The percentage of patients aged 18 years and over with schizophrenia, bipolar affective disorder and other psychoses who have a record of blood glucose or HbA1c in the preceding 12 months | NM130 |
|  |  | The percentage of patients aged 18 years and over with schizophrenia, bipolar affective disorder and other psychoses who have a record of total cholesterol: hdl ratio in the preceding 12 months | NM129 |
|  |  | The percentage of patients with a new diagnosis of depression and/or anxiety disorder in the preceding 1 April to 31 March, whose notes record an offer of referral for psychological treatment within 3 months of the date of diagnosis | NM123 |
|  |  | The percentage of patients with schizophrenia, bipolar affective disorder and other psychoses aged 25-84 (excluding those with pre-existing CHD, diabetes, stroke and/or TIA) who have had a CVD risk assessment performed in the preceding 12 months (using an assessment tool agreed with NHS England) | NM120 |
|  |  | The percentage of patients with schizophrenia, bipolar affective disorder and other psychoses who have a comprehensive care plan documented in the record, in the preceding 12 months, agreed between individuals, their family and/or carers as applicable | NM108 |
|  |  | The percentage of patients with schizophrenia, bipolar affective disorder and other psychoses who have a record of alcohol consumption in the preceding 15 months | NM15 |
|  |  | The percentage of patients with schizophrenia, bipolar affective disorder and other psychoses who have a record of blood pressure in the preceding 15 months | NM17 |
|  |  | The percentage of patients with schizophrenia, bipolar affective disorder and other psychoses who have a record of BMI in the preceding 15 months | NM16 |
|  | **Obesity** | The contractor establishes and maintains a register of patients aged 18 or over with a BMI of 25 or more in the preceding 12 months | NM128 |
|  |  | The percentage of patients with coronary heart disease, stroke or TIA, diabetes, hypertension, peripheral arterial disease, heart failure, COPD, asthma and/ or rheumatoid arthritis who have had a BMI recorded in the preceding 12 months | NM121 |
|  | **Osteoporosis** | The contractor establishes and maintains a register of patients: 1. Aged 50 or over and who have not attained the age of 75 with a record of a fragility fracture on or after 1 April 2012 and a diagnosis of osteoporosis confirmed on DXA scan, and 2. Aged 75 or over with a record of a fragility fracture on or after 1 April 2014 and a diagnosis of osteoporosis | NM29 |
|  | **Peripheral arterial disease** | The contractor establishes and maintains a register of patients with peripheral arterial disease | NM32 |
|  |  | The percentage of patients aged 79 years or under with peripheral arterial disease in whom the last blood pressure reading (measured in the preceding 12 months) is 140/90 mmHg or less | NM67 |
|  |  | The percentage of patients with peripheral arterial disease with a record in the preceding 15 months that aspirin or an alternative anti-platelet is being taken | NM33 |
|  | **Rheumatoid arthritis** | The percentage of patients aged 50-90 years with rheumatoid arthritis who have had an assessment of fracture risk using a risk assessment tool adjusted for RA in the preceding 27 months | NM57 |
|  |  | The percentage of patients with rheumatoid arthritis aged 30-84 years who have had a cardiovascular risk assessment using a CVD risk assessment tool adjusted for RA in the preceding 15 months | NM56 |
|  |  | The percentage of patients with rheumatoid arthritis who have had a face to face annual review in the preceding 15 months | NM58 |
|  |  | The practice can produce a register of all patients aged 16 years and over with rheumatoid arthritis | NM55 |
|  | **Stroke and transient ischaemic attack** | The percentage of patients aged 79 years and under with a history of stroke or TIA in whom the last blood pressure reading (measured in the preceding 12 months) is 140/90mmHg or less | NM69 |
|  |  | The percentage of patients with a stroke shown to be non-haemorrhagic, or a history of TIA, who have a record in the preceding 12 months that an anti-platelet agent, or an anti-coagulant is being taken | NM94 |
|  |  | The percentage of patients with stroke or TIA who have had influenza immunisation in the preceding 1 August to 31 March | NM140 |
|  | **Osteoarthritis** |  |  |
|  | **Chronic liver disease** |  |  |
|  | **Blindness & visual disorders** |  |  |
|  | **Deafness & auditory disorders** |  |  |

**Table S4. Final list of quality of care indicators in primary care for patients with multimorbidity**

| **Indicator Category** | **Sub-category** | **Indicator** | **SNOMED Clinical Term** | **SNOMED CT ID** |
| --- | --- | --- | --- | --- |
| Baseline clinical checks | **Blood pressure** | Has the patient's blood pressure been checked in the past 12 months (upper limb, arm - not lower limb) | Blood pressure taking assessment (procedure) | 385845001 |
|  |  |  | Measurement of blood pressure in left arm (procedure) | 990141000000106 |
|  |  |  | Measurement of blood pressure in right arm (procedure) | 990151000000109 |
|  | "Search" tab > "blood pressure" > filter by "Procedure" | <https://termbrowser.nhs.uk/?perspective=full&conceptId1=774087005&edition=uk-edition&release=v20210929&server=https://termbrowser.nhs.uk/sct-browser-api/snomed&langRefset=999000681000001101,999001251000000103> |  |  |
|  | **Serum cholesterol** | Has the patient's serum cholesterol been checked in the past 12 months | Cholesterol measurement (procedure) | 77068002 |
|  |  |  | Cholesterol content measurement (procedure) | 195861000000108 |
|  |  |  | Total cholesterol measurement (procedure) | 121868005 |
|  |  |  | High density lipoprotein cholesterol measurement (procedure) | 28036006 |
|  |  |  | Low density lipoprotein cholesterol measurement (procedure) | 113079009 |
|  | "Search" tab > "cholesterol" > filter by "Procedure" | <https://termbrowser.nhs.uk/?perspective=full&conceptId1=77068002&edition=uk-edition&release=v20210929&server=https://termbrowser.nhs.uk/sct-browser-api/snomed&langRefset=999000681000001101,999001251000000103> |  |  |
|  |  |  |  |  |
|  |  |  |  |  |
|  | **Blood sugar levels** | Has the patient's blood sugar levels been checked in the past 12 months | Glucose measurement, blood (procedure) | 33747003 |
|  | "Search" tab > "blood sugarl" / "blood glucose" > filter by "Procedure" | <https://termbrowser.nhs.uk/?perspective=full&conceptId1=33747003&edition=uk-edition&release=v20210929&server=https://termbrowser.nhs.uk/sct-browser-api/snomed&langRefset=999000681000001101,999001251000000103> |  |  |
|  |  |  |  |  |
|  | **Smoking** | Has smoking assessment been conducted in the past 12 months | Smoking assessment (procedure) | 196771000000101 |
|  |  | Has the patient been offered smoking cessation in the past 12 months | Smoking cessation education (procedure) | 225323000 |
|  | "Search" tab > "smoking" > filter by "Procedure" | <https://termbrowser.nhs.uk/?perspective=full&conceptId1=196771000000101&edition=uk-edition&release=v20210929&server=https://termbrowser.nhs.uk/sct-browser-api/snomed&langRefset=999000681000001101,999001251000000103> |  |  |
|  |  |  |  |  |
|  | **BMI** | Has the patient's Body Mass index been assessed in the past 12 months | Weight and body mass assessment procedure (procedure) | 225171007 |
|  |  |  | Measurement of body mass index (procedure) | 698094009 |
|  | **Annual influenza vaccination** | Has the patient been offered the influenza immunisation in the past 12 months | Administration of vaccine product containing only Influenza virus antigen (procedure) | 86198006 |
|  |  | Has the patient been offered the influenza immunisation in the past 12 months | Influenza immunisation for those in the 65 years and over and other at risk groups enhanced services administration (procedure) | 166311000000109 |
|  | "Search" tab > "vaccine influenza" > filter by "Procedure" | <https://termbrowser.nhs.uk/?perspective=full&conceptId1=86198006&edition=uk-edition&release=v20210929&server=https://termbrowser.nhs.uk/sct-browser-api/snomed&langRefset=999000681000001101,999001251000000103> |  |  |
|  |  |  |  |  |
|  | **Alcohol consumption** | Has the patient been screened for alcohol consumption in the past 12 months | Alcohol consumption screening (procedure) | 171208001 |
|  |  | Has the patient been offered advice on alcohol consumption in the past 12 months | Education about alcohol consumption (procedure) | 281078001 |
|  | "Search" tab > "alcohol consumption" > filter by "Procedure" | <https://termbrowser.nhs.uk/?perspective=full&conceptId1=171208001&edition=uk-edition&release=v20210929&server=https://termbrowser.nhs.uk/sct-browser-api/snomed&langRefset=999000681000001101,999001251000000103> |  |  |
|  |  |  |  |  |
| Reviewing multimorbidity severity and related risk | **Polypharmacy reviews** | Has the patients medications been reviewed in the past 12 months | Polypharmacy medication review (procedure) | 870661000000100 |
|  |  |  | Review of medication (procedure) | 182836005 |
|  |  |  | Medication review with patient (procedure) | 88551000000109 |
|  |  |  | Structured medication review (procedure) | 1239511000000100 |
|  | "Search" tab > "medication review" > filter by "Procedure"  *There are medication reviews for different conditions too individually - but ones already listed on the right here are general overall medication reviews unspecified of condition* | <https://termbrowser.nhs.uk/?perspective=full&conceptId1=171208001&edition=uk-edition&release=v20210929&server=https://termbrowser.nhs.uk/sct-browser-api/snomed&langRefset=999000681000001101,999001251000000103> |  |  |
|  | **Assessing frailty and functional assessment** | Has the patient been assessed for frailty in the past 12 months | Frailty assessment (procedure | 1086611000000106 |
|  |  |  | Assessment using frailty assessment tool (procedure) | 1092591000000101 |
|  |  |  | Frailty needs assessment (procedure) | 748111000000107 |
|  |  |  | Comprehensive frailty needs assessment (procedure) | 164091000000108 |
|  |  | Has the Electronic Frailty Index (eFI) been used to assess frailty in the patient in the past 12 months | Assessment using Electronic Frailty Index (procedure) | 1084971000000103 |
|  |  | Has a gait speed test been performed to assess functional assessment in the patient in the past 12 months | Gait speed test (procedure) | 983281000000101 |
|  |  | Has PRISMA-7 Questionnaire been conducted to assess frailty in the patient in the past 12 months (PRISMA-7 identifies disability, score of > 3 is considered to identify frailty) | Assessment using Prisma 7 Questionnaire (procedure) | 975671000000107 |
|  | "Search" tab > "frailty" > filter by "Procedure" | <https://termbrowser.nhs.uk/?perspective=full&conceptId1=171208001&edition=uk-edition&release=v20210929&server=https://termbrowser.nhs.uk/sct-browser-api/snomed&langRefset=999000681000001101,999001251000000103> |  |  |
|  | **Reviewing risk of hospital admission** | Has the patient been assessed for risk of hospital admission in the past 12 months | Assessment using QAdmissions emergency admission risk calculator (procedure) | 1099021000000103 |
|  | **Review of care plans** | Has the patient's care plans been reviewed in the past 12 months | Review of care plan (procedure) | 425268008 |
|  |  |  | Review of personal care plan (procedure) | 523031000000107 |
|  |  |  | Review of integrated care plan (procedure) | 939091000000102 |
|  |  |  | Review of supportive care plan (procedure) | 837611000000100 |
|  | **Mental health screening (Depression/anxiety)** | Has the patient been screened or assessed for mental health conditions in the past 12 months | Mental health screening (procedure) | 385892002 |
|  |  |  | Mental health screening assessment (procedure) | 410229003 |
|  |  | Has the patient been screened for depression or depressive symptoms in the past 12 months | Depression screening using Patient Health Questionnaire Nine Item score (procedure) | 715252007 |
|  |  |  | Depression screening using questions (procedure) | 200971000000100 |
|  |  | Has the patient been screened for anxiety in the past 12 months | Anxiety screening (procedure) | 908501000000101 |
|  |  |  | Anxiety screening using questions (procedure) | 908521000000105 |
|  | **Reviewing risk of falls** | Has the patient been assessed for risk of falls in the past 12 months | Screening for risk of falls (procedure) | 837561000000102 |
|  |  |  | Fall risk assessment (procedure) | 414191008 |
|  | "Search" tab > "fall risk" > filter by "Procedure" | <https://termbrowser.nhs.uk/?perspective=full&conceptId1=414191008&edition=uk-edition&release=v20210929&server=https://termbrowser.nhs.uk/sct-browser-api/snomed&langRefset=999000681000001101,999001251000000103> |  |  |

**Table S5. Patient demographic and health characteristics**

| **Characteristics** | **2015-16**  **(N=268,844)** | **2018-19**  **(n=336,693)** |
| --- | --- | --- |
| **Registered patients** | 1,563,027 | 1,479,277 |
| **Age (Mean, SD)** | 54.5 (15.7) | 55.7 (16.5) |
| < 30 years | 19,864 (7.4%) | 26,380 (7.8%) |
| 30-60 years | 141,428 (52.6%) | 163,914 (48.7%) |
| ≥60 years | 107,552 (40.0%) | 146,399 (43.5%) |
| **Gender** |  |  |
| Female | 147,976 (55.0%) | 184,751 (54.9%) |
| Male | 120,860 (45.0%) | 151,931 (45.1%) |
| Missing | 8 (<0.01%) | 11(<0.01%) |
| **Ethnicity** |  |  |
| White | 127,259 (47.3%) | 158,334 (47.0%) |
| Asian | 78,880 (29.4%) | 99,230 (29.5%) |
| Black/African-Caribbean | 31,958 (11.9%) | 38,968 (11.6%) |
| Others (inc. mixed ethnicity) | 30,198 (11.2%) | 39,434 (11.7%) |
| Missing (inc. not known/stated) | 549 (0.2%) | 727 (0.2%) |
| **Index of multiple Deprivation** (Deciles) |  |  |
| Most deprived decile | 7,991 (3.0%) | 9,788 (2.9%) |
| 2^nd^ | 6,620 (2.4%) | 8,232 (2.4%) |
| 3^rd^ | 33,536 (12.5%) | 41,577 (12.4%) |
| 4^th^ | 41,761 (15.5%) | 52,579 (15.6%) |
| 5^th^ | 44,711 (16.6%) | 56,490 (16.8%) |
| 6^th^ | 39,965 (14.9%) | 50,064 (14.9%) |
| 7^th^ | 34,355 (12.8%) | 43,003 (12.8%) |
| 8^th^ | 23,341 (8.7%) | 29,308 (8.7%) |
| 9^th^ | 21,269 (7.9%) | 26,572 (7.9%) |
| Least deprived decile | 14,740 (5.5%) | 18,362 (5.4%) |
| Missing | 555 (0.2%) | 718 (0.2%) |
| **Multimorbidity** (median, IQR) | 2 (2-3) | 2 (2-3) |
| **Number of comorbidities** (range) | (2-11) | (2-11) |
| Total with 2 morbidities | 184,746 (68.7%) | 228,979 (68.0%) |
| Total with 3 morbidities | 50,613 (18.8%) | 64,309 (19.1%) |
| Total with 4 or more morbidities | 33,485(12.5%) | 43,405 (12.9%) |
| **Hypertension** | 133,407 (49.6%) | 165,030 (49.0%) |
| **Diabetes** | 72,558 (27.0%) | 93,671 (27.8%) |
| **Polypharmacy (past 12 months)** |  |  |
| yes | 109,967 (40.9%) | 129,050 (38.3%) |
| no | 154,266 (57.4%) | 201,072 (59.7%) |
| missing | 4,611 (1.7%) | 6571 (2.0%) |
| **Frailty index categories** |  |  |
| Fit | 86,188 (32.1%) | 120,121 (35.6%) |
| Mild | 70,395 (26.2%) | 88,501 (26.3%) |
| Moderate | 59,053 (21.9%) | 68,647 (20.4%) |
| Severe | 48,597 (18.1%) | 52,853 (15.7%) |
| Missing | 4,611 (1.7%) | 6,571 (2.0%) |
| **QI scores (Median, IQR)** |  |  |
| Total QI scores | 4 (2-6) | 5 (3-6) |
| Generic indicators of clinical checks | 4 (2-5) | 4 (2-6) |
| Multimorbidity specific Indicators | 0 (0-1) | 0 (0-1) |

**Table S6. The levels of quality Indicators among multimorbidity patients in Northwest London (2015-24)**

| **Quality indicators** | **2015-16**  **(n=268,844)** | **%** | **2016-17**  **(n=285,136)** | **%** | **2017-18**  **(n=311,666)** | **%** | **2018-19**  **(n=336,693)** | **%** | **2019-20**  **(n=363,197)** | **%** |
| --- | --- | --- | --- | --- | --- | --- | --- | --- | --- | --- |
| **Generic indicators** |  |  |  |  |  |  |  |  |  |  |
| blood pressure | 206,829 | 76.9% | 225, 334 | 79.0% | 247,473 | 79.4% | 268,531 | 79.8% | 284,743 | 78.4% |
| serum cholesterol | 138,580 | 51.6% | 145,288 | 51.0% | 156,996 | 50.4% | 149,442 | 44.4% | 152,712 | 42.1% |
| blood sugar levels | 131,710 | 49.0% | 158,017 | 55.4% | 184,251 | 59.1% | 211,658 | 62.9% | 232,778 | 64.1% |
| smoking | 73,284 | 27.3% | 78,396 | 27.5% | 88,759 | 28.5% | 98,691 | 29.3% | 103, 307 | 28.4% |
| BMI | 125,314 | 46.6% | 145,295 | 51.0% | 162,378 | 52.1% | 185,665 | 55.1% | 203,456 | 56.0% |
| alcohol consumption | 81,728 | 30.4% | 90,109 | 31.6% | 97,358 | 31.2% | 104,941 | 31.2% | 110,985 | 30.6% |
| annual influenza vaccination | 122,238 | 45.5% | 144,637 | 50.7% | 174,266 | 55.9% | 189,571 | 56.3% | 190,680 | 52.5% |
| medication reviews | 88,887 | 33.1% | 100,155 | 35.1% | 110,260 | 35.4% | 127,935 | 38.0% | 136,499 | 37.6% |
| **Multimorbidity-specific indicators** |  |  |  |  |  |  |  |  |  |  |
| polypharmacy reviews | 74,649 | 27.8% | 80,245 | 28.1% | 88,410 | 28.4% | 93,055 | 27.6% | 89,948 | 24.8% |
| frailty and functional assessment | 20,785 | 7.7% | 22,069 | 7.7% | 31,652 | 10.2% | 34,616 | 10.3% | 38,959 | 10.7% |
| reviewing risk of hospital admission | 1,587 | 0.6% | 122 | 0.04% | 407 | 0.13% | 628 | 0.19% | 905 | 0.25% |
| review of care plans | 19,235 | 7.2% | 28,901 | 10.1% | 26,409 | 8.5% | 29,580 | 8.8% | 34,275 | 9.4% |
| mental health screening | 12,918 | 4.8% | 11,276 | 4.0% | 7,451 | 2.4% | 7,391 | 2.2% | 6,966 | 1.9% |
| reviewing risk of falls | 257 | 0.1% | 277 | 0.1% | 497 | 0.16% | 837 | 0.25% | 1,290 | 0.36% |

**Table S6 (continued). The levels of quality Indicators among multimorbidity patients in Northwest London (2015-24)**

| **Quality indicators** | **2020-21**  **(n=390,307)** | **%** | **2021-22**  **(n=352,779)** | **%** | **2022-23**  **(n=468,172)** | **%** | **2023-24**  **(n=509,782)** | **%** |
| --- | --- | --- | --- | --- | --- | --- | --- | --- |
| **Generic indicators** |  |  |  |  |  |  |  |  |
| blood pressure | 216,347 | 55.4% | 236,617 | 67.1% | 347,137 | 74.2% | 387,023 | 75.9% |
| serum cholesterol | 129,454 | 33.2% | 135,543 | 38.4% | 194,977 | 41.7% | 240,107 | 47.1% |
| blood sugar levels | 214,539 | 55.0% | 224,246 | 63.6% | 321,477 | 68.7% | 360,385 | 70.7% |
| smoking | 91,716 | 23.5% | 99,641 | 28.2% | 153,932 | 32.9% | 160,503 | 31.5% |
| BMI | 165,380 | 42.4% | 195,858 | 55.5% | 291,684 | 62.3% | 325,104 | 63.8% |
| alcohol consumption | 98,252 | 25.2% | 117,599 | 33.3% | 167,163 | 35.7% | 186,838 | 36.7% |
| annual influenza vaccination | 218,469 | 56.0% | 207,323 | 58.8% | 274,378 | 58.6% | 274,263 | 53.8% |
| medication reviews | 132,371 | 33.9% | 127,027 | 36.0% | 185,907 | 39.7% | 204,301 | 40.1% |
| **Multimorbidity-specific indicators** |  |  |  |  |  |  |  |  |
| polypharmacy reviews | 79,654 | 20.4% | 78,205 | 22.2% | 129,170 | 27.6% | 128,072 | 25.1% |
| frailty and functional assessment | 35,858 | 9.2% | 30,828 | 8.7% | 49,825 | 10.4% | 51,999 | 10.2% |
| reviewing risk of hospital admission | 159 | 0.04% | 187 | 0.05% | 230 | 0.05% | 228 | 0.04% |
| review of care plans | 30,966 | 7.9% | 27,821 | 7.9% | 48,828 | 10.4% | 57,714 | 11.3% |
| mental health screening | 4,562 | 1.2% | 3,705 | 1.1% | 3,849 | 0.8% | 3,535 | 0.7% |
| reviewing risk of falls | 891 | 0.23% | 1,299 | 0.37% | 2,512 | 0.54% | 4,169 | 0.82% |

**Table S7. Sociodemographic and health inequalities in individual generic quality indicators (2023-24)**

| **Characteristics** | blood pressure | serum cholesterol | blood sugar levels | smoking | BMI | alcohol consumption | annual influenza vaccination | medication reviews |
| --- | --- | --- | --- | --- | --- | --- | --- | --- |
|  | ORs (95%CI) | ORs (95%CI) | ORs (95%CI) | ORs (95%CI) | ORs (95%CI) | ORs (95%CI) | ORs (95%CI) | ORs (95%CI) |
| **Age** (ref. < 30 years) | 1.0 | 1.0 | 1.0 | 1.0 | 1.0 | 1.0 | 1.0 | 1.0 |
| 30-60 years | 2.44 (2.39-2.50) | 2.99 (2.90-3.07) | 2.60 (2.54-2.66) | 1.37 (1.33-1.40) | 1.69 (1.66-1.74) | 1.56 (1.52-1.60) | 2.07 (2.01-2.13) | 1.12 (1.09-1.15) |
| ≥60 years | 5.14 (5.01-5.28) | 3.96 (3.85-4.08) | 4.48 (4.36-4.60) | 1.10 (1.07-1.13) | 1.98 (1.93-2.03) | 1.54 (1.50-1.58) | 6.02 (5.85-6.20) | 1.16 (1.13-1.19) |
| **Gender (**ref. female) | 1.0 | 1.0 | 1.0 | 1.0 | 1.0 | 1.0 | 1.0 | 1.0 |
| Male | 1.19 (1.18-1.21) | 1.16 (1.14-1.17) | 1.17 (1.15-1.18) | 2.01 (1.99-2.04) | 1.15 (1.13-1.16) | 1.22 (1.21-1.24) | 1.26 (1.24-1.28) | 1.12 (1.10-1.13) |
| **Ethnicity** (ref. White) | 1.0 | 1.0 | 1.0 | 1.0 | 1.0 | 1.0 | 1.0 | 1.0 |
| Asian | 1.80 (1.77-1.84) | 0.92 (0.90-0.93) | 2.42 (2.38-2.46) | 0.43 (0.43-0.44) | 2.04 (2.01-2.07) | 1.71 (1.68-1.73) | 1.34 (1.32-1.36) | 0.89 (0.88-0.90) |
| Black/African-Caribbean | 1.46 (1.43-1.50) | 0.91 (0.89-0.92) | 1.59 (1.55-1.63) | 0.69 (0.67-0.70) | 1.56 (1.53-1.60) | 1.36 (1.33-1.38) | 0.92 (0.91-0.94) | 0.90 (0.88-0.92) |
| Others | 1.18 (1.15-1.20) | 1.13 (1.11-1.15) | 1.37 (1.34-1.40) | 0.75 (0.74-0.77) | 1.32 (1.29-1.34) | 1.21 (1.19-1.24) | 0.86 (0.84-0.87) | 1.03 (1.01-1.05) |
| **IMD** (ref. more deprived) | 0.99 (0.99-0.998) | 0.95 (0.95-0.96) | 0.99 (0.98-0.99) | 0.95 (0.94-0.95) | 0.98 (0.97-0.98) | 0.97 (0.97-0.98) | 1.03 (1.02-1.03) | 0.96 (0.95-0.96) |
| **Number of comorbidities** (ref. 2 morbidities) | 1.0 | 1.0 | 1.0 | 1.0 | 1.0 | 1.0 | 1.0 | 1.0 |
| 3 morbidities | 1.67 (1.64-1.71) | 1.28 (1.26-1.30) | 1.53 (1.50-1.56) | 1.39 (1.37-1.41) | 1.70 (1.68-1.73) | 1.33 (1.31-1.35) | 1.47 (1.44-1.49) | 1.48 (1.46-1.50) |
| 4 or more morbidities | 2.49 (2.40-2.58) | 1.45 (1.42-1.48) | 2.08 (2.02-2.14) | 1.71 (1.68-1.75) | 2.39 (2.33-2.45) | 1.52 (1.49-1.55) | 1.53 (1.50-1.57) | 1.90 (1.86-1.93) |
| **Polypharmacy** (ref.no) | 1.78 (1.74-1.81) | 1.21 (1.20-1.23) | 1.63 (1.60-1.66) | 1.32 (1.29-1.34) | 1.47 (1.45-1.49) | 1.16 (1.14-1.18) | 1.55 (1.53-1.58) | 1.30 (1.28-1.32) |
| **Frailty** (ref. fit) | 1.0 | 1.0 | 1.0 | 1.0 | 1.0 | 1.0 | 1.0 | 1.0 |
| Mild | 1.75 (1.71-1.78) | 1.33 (1.31-1.35) | 1.74 (1.71-1.77) | 1.22 (1.20-1.24) | 1.44 (1.41-1.46) | 1.29 (1.27-1.31) | 1.70 (1.68-1.73) | 1.32 (1.30-1.34) |
| Moderate | 2.29 (2.22-2.36) | 1.29 (1.26-1.31) | 2.13 (2.08-2.19) | 1.17 (1.14-1.19) | 1.59 (1.55-1.62) | 1.48 (1.45-1.51) | 2.05 (2.01-2.10) | 1.47 (1.44-1.50) |
| Severe | 2.92 (2.78-3.05) | 1.12 (1.09-1.15) | 2.13 (2.06-2.21) | 1.02 (0.99-1.05) | 1.50 (1.45-1.54) | 1.75 (1.71-1.79) | 1.86 (1.81-1.91) | 1.93 (1.88-1.98) |

*Note*. Models adjusted for age, gender, ethnicity, IMD, number of comorbidities, polypharmacy, and frailty index. Odds ratios: OR; CI: Confidence Intervals.

**Table S8. Sociodemographic and health inequalities in individual multimorbidity specific quality indicators (2023-24)**

| **Characteristics** | polypharmacy reviews | frailty and functional assessment | reviewing risk of hospital admission | review of care plans | mental health screening | reviewing risk of falls |
| --- | --- | --- | --- | --- | --- | --- |
|  | ORs (95%CI) | ORs (95%CI) | ORs (95%CI) | ORs (95%CI) | ORs (95%CI) | ORs (95%CI) |
| **Age** (ref. < 30 years) | 1.0 | 1.0 | 1.0 | 1.0 | 1.0 | 1.0 |
| 30-60 years | 1.43 (1.38-1.48) | 2.95 (2.55-3.41) | 1.74 (0.63-4.76) | 0.97 (0.93-1.02) | 0.56 (0.50-0.62) | 0.66 (0.50-0.86) |
| ≥60 years | 1.72 (1.66-1.78) | 9.53 (8.24-11.0) | 0.61 (0.22-1.71) | 0.96 (0.91-1.00) | 0.31 (0.27-0.35) | 1.10 (0.84-1.43) |
| **Gender (**ref. female) | 1.0 | 1.0 | 1.0 | 1.0 | 1.0 | 1.0 |
| Male | 1.12(1.10-1.13) | 1.03 (1.01-1.05) | 2.13 (1.64-2.79) | 1.07 (1.05-1.09) | 0.56 (0.52-0.60) | 1.22 (1.14-1.30) |
| **Ethnicity** (ref. White) | 1.0 | 1.0 | 1.0 | 1.0 | 1.0 | 1.0 |
| Asian | 1.07 (1.05-1.09) | 0.95 (0.93-0.98) | 1.56 (1.12-2.18) | 0.67 (0.66-0.69) | 1.26 (1.17-1.37) | 0.65 (0.61-0.70) |
| Black/African-Caribbean | 1.10 (1.07-1.12) | 0.92 (0.89-0.95) | 2.97 (2.09-4.21) | 0.95 (0.92-0.98) | 0.92 (0.82-1.03) | 0.92 (0.83-1.02) |
| Others | 0.84 (0.82-0.86) | 1.03 (1.00-1.07) | 0.90 (0.53-1.52) | 1.07 (1.01-1.10) | 0.95 (0.86-1.06) | 0.76 (0.68-0.85) |
| **IMD** (ref. more deprived) | 1.05 (1.04-1.05) | 0.96 (0.96-0.97) | 0.76 (0.71-0.81) | 0.94 (0.94-0.95) | 0.98 (0.96-0.99) | 0.97 (0.96-0.98) |
| **Number of comorbidities** (ref. 2 morbidities) | 1.0 | 1.0 | 1.0 | 1.0 | 1.0 | 1.0 |
| 3 morbidities | 1.18 (1.16-1.20) | 1.15 (1.12-1.18) | 1.02 (0.73-1.42) | 1.48 (1.44-1.51) | 1.04 (0.96-1.14) | 0.93 (0.86-1.01) |
| 4 or more morbidities | 1.30 (1.27-1.32) | 1.28 (1.25-1.32) | 1.14 (0.81-1.59) | 1.67 (1.63-1.71) | 1.27 (1.14-1.41) | 0.90 (0.83-0.97) |
| **Polypharmacy** (ref.no) | 1.67 (1.64-1.69) | 0.91 (0.89-0.93) | 1.27 (0.93-1.74) | 0.91 (0.89-0.93) | 1.67 (1.54-1.81) | 1.39 (1.29-1.51) |
| **Frailty** (ref. fit) | 1.0 | 1.0 | 1.0 | 1.0 | 1.0 | 1.0 |
| Mild | 1.67 (1.64-1.70) | 2.80 (2.68-2.92) | 5.65 (3.20-9.98) | 1.59 (1.55-1.64) | 1.36 (1.24-1.48) | 2.98 (2.50-3.56) |
| Moderate | 2.26 (2.21-2.31) | 8.69 (8.33-9.06) | 15.57 (8.69-27.89) | 2.94 (2.85-3.04) | 1.28 (1.14-1.44) | 8.27 (6.97-9.82) |
| Severe | 2.91 (2.84-2.99) | 22.50 (21.50-23.55) | 31.55 (16.72-59.52) | 7.96 (7.68-8.25) | 1.38 (1.19-1.59) | 35.81 (30.11-42.60) |

*Note*. Models adjusted for age, gender, ethnicity, IMD, number of comorbidities, polypharmacy, and frailty index. Odds ratios: OR; CI: Confidence Intervals.

**Sensitivity analysis**

**Table S9. Sociodemographic and health inequalities in individual generic quality indicators (2018-19)**

| **Characteristics** | blood pressure | serum cholesterol | blood sugar levels | Smoking | BMI | alcohol consumption | annual influenza vaccination | medication reviews |
| --- | --- | --- | --- | --- | --- | --- | --- | --- |
|  | ORs (95%CI) | ORs (95%CI) | ORs (95%CI) | ORs (95%CI) | ORs (95%CI) | ORs (95%CI) | ORs (95%CI) | ORs (95%CI) |
| **Age** (ref. < 30 years) | 1.0 | 1.0 | 1.0 | 1.0 | 1.0 | 1.0 | 1.0 | 1.0 |
| 30-60 years | 2.01 (1.95-2.07) | 4.43 (4.26-4.62) | 3.17 (3.07-3.27) | 1.15 (1.12-1.19) | 1.31 (1.28-1.35) | 1.11 (1.08-1.15) | 2.11 (2.04-2.19) | 1.27 (1.23-1.31) |
| ≥60 years | 3.84 (3.71-3.97) | 6.10 (5.85-6.36) | 4.83 (4.67-5.00) | 0.76 (0.73-0.79) | 1.32 (1.28-1.36) | 0.92 (0.89-0.95) | 6.64 (6.40-6.89) | 1.48 (1.43-1.53) |
| **Gender** (ref. female) | 1.0 | 1.0 | 1.0 | 1.0 | 1.0 | 1.0 | 1.0 | 1.0 |
| Male | 1.13 (1.11-1.15) | 1.20 (1.19-1.22) | 1.22 (1.20-1.24) | 2.01 (1.98-2.04) | 1.11 (1.10-1.13) | 1.22 (1.20-1.24) | 1.42 (1.39-1.44) | 1.05 (1.04-1.07) |
| **Ethnicity** (ref. White) | 1.0 | 1.0 | 1.0 | 1.0 | 1.0 | 1.0 | 1.0 | 1.0 |
| Asian | 1.83 (1.79-1.87) | 1.20 (1.18-1.22) | 2.66 (2.61-2.71) | 0.39 (0.39-0.40) | 1.76 (1.73-1.79) | 1.39 (1.37-1.42) | 1.74 (1.70-1.77) | 0.99 (0.97-1.01) |
| Black/African-Caribbean | 1.54 (1.50-1.59) | 1.17 (1.14-1.20) | 1.71 (1.67-1.75) | 0.61 (0.60-0.63) | 1.43 (1.40-1.46) | 1.14 (1.11-1.17) | 1.19 (1.16-1.22) | 0.88 (0.86-0.90) |
| Others | 1.17 (1.14-1.21) | 1.31 (1.28-1.34) | 1.53 (1.49-1.56) | 0.72 (0.70-0.74) | 1.30 (1.27-1.33) | 1.22 (1.19-1.25) | 0.96 (0.93-0.98) | 1.09 (1.07-1.12) |
| **IMD** | 0.99 (0.99-1.00) | 0.96 (0.95-0.96) | 0.98 (0.98-0.99) | 0.95 (0.95-0.95) | 0.96 (0.96-0.97) | 0.97 (0.96-0.97) | 1.02 (1.02-1.02) | 0.97 (0.97-0.97) |
| **Number of comorbidities**  (ref. 2 morbidities) | 1.0 | 1.0 | 1.0 | 1.0 | 1.0 | 1.0 | 1.0 | 1.0 |
| 3 morbidities | 1.76 (1.71-1.81) | 1.22 (1.20-1.24) | 1.44 (1.41-1.47) | 1.37 (1.34-1.40) | 1.52 (1.49-1.55) | 1.36 (1.33-1.39) | 1.85 (1.81-1.89) | 1.37 (1.34-1.39) |
| 4 or more morbidities | 3.16 (3.00-3.33) | 1.37 (1.34-1.40) | 2.01 (1.95-2.07) | 1.68 (1.64-1.72) | 2.13 (2.08-2.19) | 1.67 (1.63-1.71) | 2.82 (2.73-2.91) | 1.62 (1.59-1.66) |
| **Polypharmacy** (ref.no) | 1.18 (1.15-1.21) | 1.09 (1.07-1.11) | 1.15 (1.12-1.17) | 1.23 (1.20-1.25) | 1.10 (1.08-1.12) | 0.95 (0.93-0.97) | 1.33 (1.30-1.36) | 1.11 (1.09-1.13) |
| **Frailty** (ref. fit) | 1.0 | 1.0 | 1.0 | 1.0 | 1.0 | 1.0 | 1.0 |  |
| Mild | 1.82 (1.78-1.86) | 1.43 (1.40-1.46) | 1.66 (1.63-1.70) | 1.16 (1.13-1.18) | 1.34 (1.32-1.37) | 1.17 (1.14-1.19) | 1.88 (1.84-1.92) | 1.23 (1.21-1.26) |
| Moderate | 2.56 (2.48-2.65) | 1.52 (1.49-1.56) | 2.05 (1.99-2.10) | 1.17 (1.14-1.20) | 1.51 (1.47-1.54) | 1.28 (1.25-1.31) | 2.66 (2.59-2.72) | 1.35 (1.32-1.38) |
| Severe | 3.94 (3.75-4.15) | 1.50 (1.46-1.54) | 2.44 (2.36-2.52) | 1.10 (1.06-1.13) | 1.67 (1.62-1.72) | 1.61 (1.56-1.66) | 3.29 (3.18-3.41) | 1.64 (1.59-1.68) |

*Note*. Models adjusted for age, gender, ethnicity, IMD, number of comorbidities, polypharmacy, and frailty index.

**Table S10. Sociodemographic and health inequalities in individual multimorbidity specific quality indicators and overall scores of quality indicators (2018-19)**

| **Characteristics** | polypharmacy reviews | Frailty and functional assessment | reviewing risk of hospital admission | review of care plans | mental health screening | reviewing risk of falls | Overall score  (Coef.) |
| --- | --- | --- | --- | --- | --- | --- | --- |
|  | ORs (95%CI) | ORs (95%CI) | ORs (95%CI) | ORs (95%CI) | ORs (95%CI) | ORs (95%CI) | β |
| **Age** (ref. < 30 years) | 1.0 | 1.0 | 1.0 | 1.0 | 1.0 | 1.0 | 1.0 |
| 30-60 years | 1.11 (1.07-1.15) | 2.61 (2.23-3.07) | 1.65 (0.66-4.10) | 0.86 (0.81-0.91) | 0.95 (0.84-1.08) | 1.26 (0.46-3.48) | 0.74 (0.72-0.77) |
| ≥60 years | 1.24 (1.19-1.29) | 8.25 (7.03-9.68) | 9.01 (3.68-22.08) | 0.69 (0.65-0.73) | 0.93 (0.82-1.05) | 2.82 (1.03-7.73) | 1.14 (1.12-1.17) |
| **Gender (**ref. female) | 1.0 | 1.0 | 1.0 | 1.0 | 1.0 | 1.0 | 1.0 |
| Male | 1.05 (1.03-1.06) | 0.89 (0.87-0.91) | 1.04 (0.88-1.22) | 1.03 (1.00-1.05) | 1.22 (1.16-1.28) | 0.94 (0.82-1.09) | 0.35 (0.33-0.36) |
| **Ethnicity** (ref. White) | 1.0 | 1.0 | 1.0 | 1.0 | 1.0 | 1.0 | 1.0 |
| Asian | 1.27 (1.25-1.29) | 1.15 (1.11-1.18) | 0.38 (0.31-0.47) | 0.76 (0.73-0.78) | 1.87 (1.78-1.97) | 0.76 (0.65-0.89) | 0.30 (0.28-0.31) |
| Black/African-Caribbean | 1.27 (1.24-1.30) | 1.04 (1.00-1.09) | 0.47 (0.35-0.63) | 1.06 (1.02-1.10) | 1.14 (1.05-1.24) | 1.02 (0.82-1.28) | 0.17 (0.15-0.19) |
| Others | 0.76 (0.73-0.78) | 1.20 (1.15-1.25) | 0.29 (0.20-0.43) | 1.20 (1.15-1.24) | 0.87 (0.79-0.95) | 0.70 (0.53-0.92) | 0.11 (0.09-0.13) |
| **IMD** | 1.07 (1.07-1.08) | 0.99 (0.98-0.99) | 0.81 (0.78-0.84) | 0.94 (0.93-0.94) | 1.09 (1.07-1.10) | 0.93 (0.90-0.96) | -0.03 (-0.03 - -0.03) |
| **Number of comorbidities** (ref. 2 morbidities) | 1.0 | 1.0 | 1.0 | 1.0 | 1.0 | 1.0 | 1.0 |
| 3 morbidities | 1.07 (1.05-1.09) | 1.21 (1.17-1.25) | 1.11 (0.91-1.35) | 1.63 (1.58-1.68) | 1.30 (1.22-1.38) | 1.03 (0.86-1.23) | 0.61 (0.59-0.63) |
| 4 or more morbidities | 1.01 (0.98-1.03) | 1.51 (1.47-1.56) | 1.03 (0.83-1.26) | 2.16 (2.09-2.24) | 1.62 (1.52-1.73) | 1.24 (1.05-1.46) | 0.99 (0.96-1.01) |
| **Polypharmacy** (ref.no) | 1.40 (1.38-1.43) | 0.84 (0.82-0.87) | 1.86 (1.53-2.27) | 0.88 (0.85-0.91) | 1.16 (1.09-1.22) | 1.33 (1.11-1.60) | 0.22 (0.21-0.24) |
| **Frailty** (ref. fit) | 1.0 | 1.0 | 1.0 | 1.0 | 1.0 | 1.0 | 1.0 |
| Mild | 1.50 (1.46-1.53) | 3.62 (3.40-3.85) | 1.92 (1.38-2.68) | 1.20 (1.15-1.25) | 1.73 (1.61-1.87) | 2.33 (1.26-4.31) | 0.64 (0.63-0.66) |
| Moderate | 1.83 (1.78-1.88) | 7.80 (7.33-8.29) | 2.17 (1.54-3.07) | 1.80 (1.73-1.88) | 2.14 (1.97-2.33) | 7.98 (4.54-14.02) | 0.98 (0.96-1.00) |
| Severe | 1.93 (1.87-1.99) | 19.80 (18.57-21.11) | 2.73 (1.90-3.92) | 4.64 (4.42-4.87) | 2.60 (2.36-2.86) | 44.32 (25.33-77.52) | 1.45 (1.42-1.47) |

*Note*. Models adjusted for age, gender, ethnicity, IMD, number of comorbidities, polypharmacy, and frailty index.
